# Investigation of anthrax in an endemic region in Kenya: a mixed methods approach

**DOI:** 10.11604/pamj.supp.2018.30.1.15279

**Published:** 2018-05-18

**Authors:** Mark Odhiambo Obonyo, Mikayla Farr, Emmanuel Hikufe Hikufe, Wilson Rubanzana, Maurice Omondi Owiny, Zeinab Gura Roka

**Affiliations:** 1 Kenya Field Epidemiology and Laboratory Training Program, Kenya; 2 Rollins School of Public Health, Emory University, USA; 3 Nambia Field Epidemiology and Laboratory Training Program, Nambia; 4 School of Public Health, College of Medicine and Health Sciences, University of Rwanda, Rwanda

**Keywords:** Outbreak investigation, anthrax, Kenya, mixed methods

## Abstract

In Kenya, human anthrax cases most often occur linked to animal anthrax. In most cases, human behaviors, especially slaughter and consumption of meat from animal anthrax cases, has been implicated. This case study is based on an anthrax outbreak investigation conducted in an endemic region in Kenya in May 2016.The case study simulates how a mixed methods approach can be used in epidemiologic research.To fully benefit from this case study, participants should have had prior lectures or other instruction in quantitative and qualitative study designs and sampling approachesused in epidemiologic research. The case study is ideally suited for trainees at intermediate or advance level training in field epidemiology who should be able to complete the case study in approximately 3 hours.

## How to use this Study

**General instructions:** the case study is suited for a class of up to 20 trainees per session. Ideally, 1 to 2 instructors can facilitate the case study in a classroom or conference room. The instructor facilitating the session should direct participants to read a paragraph out aloud, going around the room to give each participant a chance to read. When a participant reads a question, the instructor may choose to engage the class in large group discussion of the answer, randomly identify a participant to respond to the question, or divide the class into smaller groups for exercises, depending on the type of question. The role of the instructor is largely to coordinate the session such that participants learn more from each other, and not just from the instructor.

**Audience:** intermediate and advance level trainees in the Field Epidemiology and Laboratory Training Program (FELTP).

**Prerequisites:** for this case study, participants should have received lectures or other instructions in; Study designs in epidemiologic research; Survey and sampling in epidemiologic research; Ethics in Research: Writing an Informed Consent Form.

**Materials needed:** flipcharts or white board with markers.

**Level of training:** intermediate and advanced level training in epidemiology.

**Time required:** approximately 3 hours

**Language:** English

## Competing interest

The authors declare no competing interest.
